# Delayed Diagnosis of Whipple’s Disease Complicated by Jarisch–Herxheimer Reaction to Ceftriaxone Treatment: A Case Report and Literature Review

**DOI:** 10.3390/tropicalmed7030040

**Published:** 2022-03-03

**Authors:** Marcus C. C. Clarke, Ric N. Price

**Affiliations:** 1 Division of Medicine, Royal Darwin Hospital, 105 Rocklands Dr, Tiwi, NT 0810, Australia; ric.price@menzies.edu.au; 2 Global and Tropical Health Division, Menzies School of Health Research, Charles Darwin University, Ellengowan Dr, Casuarina, NT 0810, Australia; 3 Centre for Tropical Medicine and Global Health, Nuffield Department of Clinical Medicine, University of Oxford, Oxford OX1 2JD, UK

**Keywords:** Whipple’s disease, Jarisch–Herxheimer reaction, antimicrobial treatment

## Abstract

Whipple’s disease is a rare chronic infection caused by the actinomycete *Tropheryma whipplei.* Patients commonly present with gastrointestinal symptoms. We present a case of classic Whipple’s disease complicated by a probable Jarisch–Herxheimer reaction following the initiation of ceftriaxone treatment.

## 1. Introduction

Whipple’s disease (WD) is a multi-system disorder caused by infection with the ubiquitous environmental actinomycete *Tropheryma whipplei,* first described by George Hoyt Whipple in 1907 [1]. The diagnosis is rare, with an estimated global prevalence of two per million population [2]. If left untreated, WD is potentially fatal, with an estimated overall mortality of 10%, but with early diagnosis and antibiotic treatment the response is usually rapid [3,4].

We present a case of a 43-year-old male with classic WD who had a typically delayed diagnosis and a probable Jarisch–Herxheimer reaction (JHR) following the initiation of antimicrobial therapy. The latter is an adverse event that usually commences within 24 h of starting antibiotic therapy, manifesting as fever, chills, headache, myalgia, and intensification of skin rashes [5] We present a review of the literature on this adverse reaction and its association with WD.

## 2. Case Description

A 43-year-old man was referred to the infectious disease team at the Royal Darwin Hospital following an upper gastrointestinal endoscopy. The patient was an Australian-born horticultural engineer living in an urban area in northern Australia. He was of European ancestry, had no prior medical history, no medication allergies, and had lived in the Northern Territory of Australia for 13 years. He had taken several short holidays in Indonesia, Cambodia, and Vietnam. His only regular medication was finasteride, which was used for androgenetic alopecia.

The patient described an 8-year history of fluctuating and often debilitating malaise, but did not develop gastrointestinal symptoms until the third year of his illness. His initial symptoms included monthly episodes of subjective fever, chills, and extreme lethargy lasting 24 h, which resolved spontaneously. Investigations at the time included blood cultures, thick and thin blood films for malaria, urinalysis, and chest radiography, all of which were normal. The erythrocyte sedimentation rate was 25 mm/h (normal: <10 mm/h), and the ferritin level was elevated to 508 µg/L (normal range: 20–220 µg/L). Despite the lack of gastrointestinal symptoms, microscopic stool examination was undertaken and revealed *Giardia lamblia* cysts. He was treated with a single dose of tinidazole, but showed no improvement in his episodic malaise. In the second year of his illness, he developed arthralgia of the hands, wrists, elbows, shoulders, and knees, which was associated with morning stiffness but no evidence of arthritis. His C-reactive protein (CRP) level was 14 mg/L (normal: <5 mg/L). An autoantibody screen for inflammatory arthritis tested negative. The patient was managed with paracetamol and nonsteroidal anti-inflammatory medications.

By the third year of his illness, the patient’s episodic subjective fevers had resolved, but he had developed non-bloody diarrhoea (watery bowel motions up to 8 times per day) without abdominal pain and had lost 15 kg of body weight. Investigations revealed low hydroxocobalamin, 165 pmol/L (normal range: 200–900 pmol/L), and faecal examination once again revealed *G. lamblia* cysts. A faecal calprotectin test was not performed. He was prescribed a 3-day course of metronidazole, but his symptoms did not improve. Computed tomography of the chest, abdomen, and pelvis revealed mildly enlarged mesenteric lymph nodes, but no other significant findings.

In the eighth year of his illness, upper and lower gastrointestinal endoscopy was performed to investigate the persistent diarrhoea. The macroscopic appearance of the duodenum was consistent with duodenitis, and microscopic examination of the duodenal tissue revealed macrophages within the lamina propria with periodic acid-Schiff (PAS)-positive intracytoplasmic inclusions consistent with WD. The histopathological findings prompted subsequent testing for *T. whipplei* 16S rRNA polymerase chain reaction (PCR), which confirmed the presence of the organism in biopsy specimens.

After confirmation of a diagnosis of WD, the patient was referred to the infectious diseases unit. On review, the patient appeared well, with a heart rate of 80 beats per minute, blood pressure of 110/60 mmHg, and a tympanic temperature of 37.0 °C. His body mass index was 21, and he had bilateral quadriceps wasting and hyperpigmentation of the skin over the umbilicus. The clinical examination was otherwise unremarkable. Blood investigations revealed mild normocytic anaemia, with a haemoglobin level of 129 g/L (normal range: 135–185 g/L). Total protein level was 58 g/L (normal range: 64–84 g/L), and CRP level was 21 mg/L. HIV antigen/antibody testing was nonreactive. Due to the association of *T. whipplei* with disseminated infection, including neurologic infection and endocarditis, an examination of the cerebrospinal fluid was performed [3,6,7]. This showed a white cell count of 2 × 10^6^ (normal: ≤5), red cell count of 6 × 10^6^, protein level of 0.33 g/L (normal range: 0.15–0.45 g/L), and glucose level of 3.6 mmol/L (normal range: 2.7–4.2 mmol/L); *T. whipplei* was not detected by PCR. There was no evidence of endocarditis on transthoracic echocardiography.

The patient was started on intravenous ceftriaxone 2 g daily. Within 7 h of administering the first dose, he developed marked nausea, vomiting, chills, and sweats, but no rash or urticaria. His temperature rose to 37.7 °C, which peaked at 38.8 °C four hours later. His symptoms resolved completely overnight, without any medical intervention. On further questioning, he denied similar prior episodes or a history of hypersensitivity to cephalosporins or related medications. 

The patient was administered ceftriaxone 2 g daily for two weeks, without any additional adverse effects, followed by oral trimethoprim–sulfamethoxazole 160/800 mg twice daily. His diarrhoea improved significantly within one week of starting antibiotics, and on review at 2 months, his gastrointestinal symptoms and arthralgia had resolved, and he had gained 12 kg in weight. At 12 months, the patient was asymptomatic, and his antibiotics were ceased.

## 3. Discussion

Classic WD presents with arthralgia, diarrhoea, abdominal pain, and weight loss. Diagnosis requires the biopsy of infected tissue, is established by identifying characteristic histopathological changes with PAS-stained macrophages, and confirmed by the presence of *T. whipplei* detected by PCR [2,3,6]. Infection often involves multiple organ systems, including the gastrointestinal, cardiovascular, neurological, and pulmonary systems [2,3]. Localised infections, self-limiting infections, and asymptomatic carriage are also described [3]. *T. whipplei* is widely distributed in the environment and has been found in the saliva and faeces of asymptomatic individuals; it is estimated to occur in 4% of Europeans [4,7,8]. The risk of asymptomatic carriage is higher in sewage workers, and is estimated to be between 12 and 20%, suggestive of faecal–oral transmission [4,9].

The optimal treatment for WD is unknown, but an initial period of intravenous antimicrobials for 2 weeks followed by prolonged oral therapy is recommended [10]. During therapy for WD, immune reconstitution inflammatory syndromes have been documented [2,5,11]. A case of probable JHR has been described in a patient treated with oral trimethoprim–sulfamethoxazole and intravenous streptomycin [5]. WD relapses are not common, but when they do occur, typically involve the central nervous system, with recurrences often occurring many years after the completion of antimicrobial therapy [2]. The non-specific nature of symptoms, the rarity of the disease, and the difficulty of diagnosis often result in delays in diagnosis and treatment. Failure to diagnose and treat WD can result in nutritional deficiencies, sepsis, irreversible brain damage, and death [2]. Indeed, prior to recognition and effective antimicrobial treatment, WD was universally fatal [5,12,13]. However, with prompt diagnosis and the initiation of treatment, the prognosis is excellent, with patients improving within 2–3 weeks of starting antibiotics [2,6,14].

*T. whipplei* is susceptible to a range of antimicrobials, including penicillin, ceftriaxone, carbapenems, trimethoprim–sulfamethoxazole, and doxycycline. Central nervous system involvement occurs in up to 40% of patients with WD; thus, antibiotics that can achieve high concentrations in the central nervous system are desirable [6,15]. Relapse of WD can occur years after treatment; hence, therapy is generally continued for at least 12 months [2,10,16].

Our case highlights the challenges in diagnosing WD, even after extensive infectious disease and rheumatological investigations. Our patient initially presented with non-specific symptoms, with arthralgia beginning after 12 months and gastrointestinal symptoms developing only in the third year of illness. It took 8 years from symptom onset to deliver the final diagnosis and commence effective therapy. This course is consistent with other studies, reporting 6 to 8 years from the first symptoms to diagnosis [4,17].

*G. lamblia* was isolated from the patient’s faeces on two separate occasions, but treatment with nitroimidazoles did not improve his symptoms. A previous study has reported that *Giardia* is present in 16% of patients with WD, which is significantly higher than in patients without WD (0.6%) [18].

Following the initiation of intravenous ceftriaxone, our patient described an acute febrile reaction which resolved spontaneously. Although an allergy to ceftriaxone was considered, there were no other features suggestive of beta-lactam hypersensitivity, and further administration of ceftriaxone was well tolerated. The most likely explanation for these symptoms is a JHR. JHR is most commonly reported in patients with spirochaete infections following the commencement of intravenous penicillin [11]. In 1992, Playford et al. reported that a patient with WD became confused, febrile, and developed retinal vasculitis 12 h after the initiation of antibiotic therapy [5]. The patient was treated with prednisolone and carbamazepine, improved while on antibiotics, and was diagnosed with a JHR [5].

It is possible that a JHR may often go unrecognised, because symptoms may be associated with the underlying infection, and exacerbation may be attributed to the disease rather than a JHR [11]. A JHR is rarely fatal and is transient, lasting no more than several hours [11]. Importantly, clinicians should consider a JHR because erroneously ascribing the symptoms to a drug allergy would result in discontinuation of first-line antimicrobial therapy.

## 4. Conclusions

Although uncommon, WD is a serious chronic infection that is difficult to diagnose but generally responds quickly to appropriate antibiotics. JHR is a potential complication of WD that should be considered in patients with worsening symptoms within 4–6 h of starting antibiotics and, in most cases, can be managed with supportive care.

## Data Availability

Not applicable.
